# Aorto-Right Ventricular Shunt after TAVR: Rare Complication of Common Procedure

**DOI:** 10.1155/2017/1834394

**Published:** 2017-09-28

**Authors:** Abdelkader Almanfi, Ahmad Qurie, Neil Strickman

**Affiliations:** Department of Cardiology, Texas Heart Institute, Houston, TX, USA

## Abstract

**Background:**

The primary treatment of symptomatic aortic stenosis is aortic valve replacement. Instead of open chest surgery, transcatheter aortic valve replacement (TAVR) is an alternative intervention for high-risk surgical candidates.

**Clinical Case:**

A 92-year-old male presented with progressive exertional dyspnea and recurrent syncopal attacks secondary to severe AS. The patient underwent successful transfemoral TAVR with 29 mm Edwards SAPIEN XT valve. His postoperative course was complicated by aorto-right ventricular shunt. The patient's clinical course was followed up for one year.

**Conclusion:**

This case reports the incidence and clinical course of one of the rare complications of TAVR, aorto-right ventricular fistula. Conservative medical management is appropriate in hemodynamically stable patients with this specific complication.

## 1. Introduction

Aortic valve replacement is still the mainstay of treatment of symptomatic aortic stenosis (AS). In properly selected patients, this surgical procedure offers substantial improvements in symptoms and life expectancy. Transcatheter aortic valve replacement (TAVR) is a procedure for patients with severe symptomatic aortic stenosis who are not candidates for traditional open-chest surgery or are high-risk operable candidates [1]. Our case describes a 92-year-old male who presented with progressive exertional dyspnea and recurrent syncopal attacks secondary to severe AS. The patient underwent successful transfemoral TAVR with 29 mm Edwards SAPIEN XT valve; his postoperative course was complicated by aorto-right ventricular fistula.

## 2. Case Report

Our patient is a 92-year-old male with severe aortic stenosis whose past medical history includes umbilical and inguinal hernia, chronic diastolic heart failure, right bundle branch block, coronary artery disease, and obesity. He became significantly symptomatic in the last few months prior to his admission to the hospital, where he complained of worsened dyspnea on exertion and recurrent syncopal attacks. Physical examination did not show any significant findings except for a 5/6 systolic ejection murmur in the aortic area radiating to both carotids. Diagnostic cardiac catheterization showed no evidence of coronary artery disease. Carotid Doppler showed no significant stenosis. Electrocardiogram showed sinus rhythm with a right bundle branch block. Transesophageal echocardiography showed critical aortic valve stenosis, mean pressure gradient of approximately 72 mmHg, valve area of 0.46 cm^2^, and left ventricular ejection fraction of 60%. A 29 mm Edwards SAPIEN XT valve was implanted via transfemoral access. Confirmation of position was then done by aortography. The transesophageal echocardiogram confirmed the adequate position of the valve with minimal aortic insufficiency. There was no pericardial effusion with normal ventricular systolic function. Postdeployment aortogram showed paravalvular leak and patent coronary arteries.

Postdeployment hemodynamics showed zero mmHg mean gradient pressure between the left ventricle and the aorta with left ventricular end diastolic pressure (LVEDP) ~ 10 mmHg.

**Figure 1:**
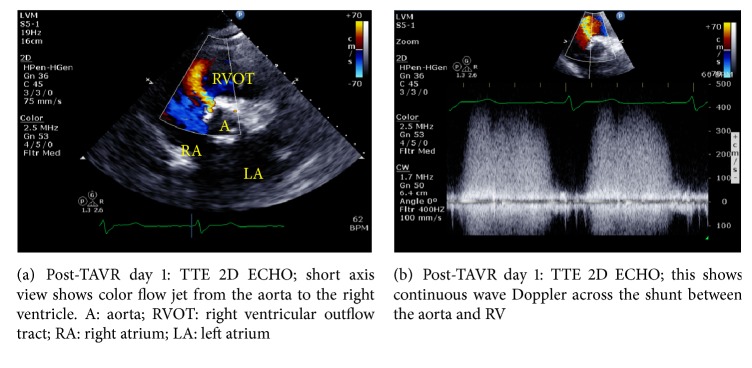


Post-TAVR day 1 routine transthoracic echocardiography (TTE) showed well-seated and normally functioning bioprosthetic aortic valve with a small posterior paravalvular leak. It also showed a small-sized continuous (systolic and diastolic phases) shunt, which communicated with the aortic root into the right ventricle (aorto-RV shunt) (Figures 1(a) and 1(b)).

**Figure 2:**
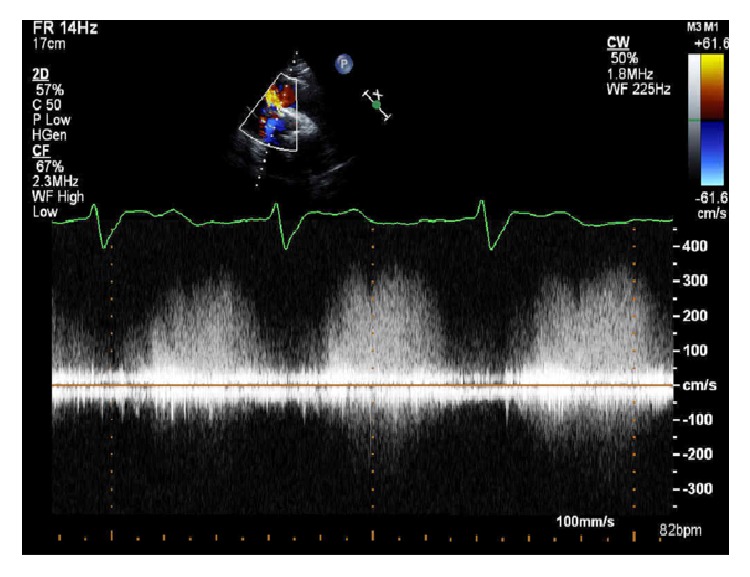
1-Month follow-up: TTE 2D ECHO; this shows continuous wave Doppler across the shunt between the aorta and RV.

Since the patient was asymptomatic following the procedure, he was managed conservatively with close follow-up. His repeat TTE a month after the procedure revealed no significant change from his post-TAVR day 1 TTE (Figure 2). Therefore, we decided to continue with medical management and close follow-up, since the patient was asymptomatic.

**Figure 3:**
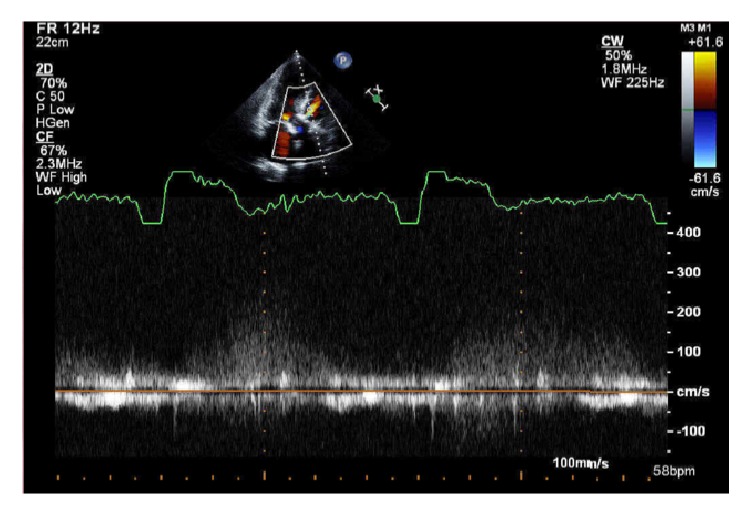
1-Year follow-up: TTE 2D ECHO with color flow shows the residual aorto-RV shunt with a continuous wave Doppler, which demonstrates significant reduction in the velocity compared to the initial measurements.

Follow-up with TTE a year later showed persistence of the aorto-RV fistula and significant improvement in the velocity across the shunt (Figure 3), which may be due to a decrease in the fistula size rather than an increase in RV pressure as RV systolic pressure (RVSP) remained within normal limits on follow-up TTE.

## 3. Discussion

A few cases of an aorto-right ventricular shunt after TAVR procedure have been reported. Complications associated with TAVR may be specific to TAVR, such as valve malposition, paravalvular aortic regurgitation, conduction abnormalities, and coronary obstruction, or not specific to TAVR, such as vascular access complications and cardiac perforation or tamponade seen with other endovascular interventions [2, 3]. In our patient, we believe that aortic root rupture may have resulted from displacement of the calcified tissue, causing local trauma. The clinical presentation of an aorto-right ventricular shunt depends on the size and hemodynamic significance of the shunt [4]. Despite the presence of the shunt, our patient was clinically asymptomatic even after one year following the procedure. However, the mean interval between the time of injury and definitive vascular repair was 1.5 years due to development of severe heart failure symptoms as reported by Samuels et al. [5]. As spontaneous closure of aorto-RV fistula has not been reported, it is very important to follow up with all patients regularly. With all the advances in interventional cardiology, successful percutaneous closure of aorto-RV fistula has been reported using a septal occluder [6].
